# Circulating Tumor Cells in Neuroblastoma

**DOI:** 10.4274/tjh.2017.0199

**Published:** 2017-12-01

**Authors:** Mili Jain, Ashutosh Kumar, Sanjay Mishra, Nishant Verma, Madhu Mati Goel

**Affiliations:** 1 King George’s Medical University, Department of Pathology, Uttar Pradesh, India; 2 King George’s Medical University, Department of Pediatrics, Uttar Pradesh, India

**Keywords:** Neuroblastoma, Circulating tumor cells, Metastasis

## To The Editor,

**Figure 1:**
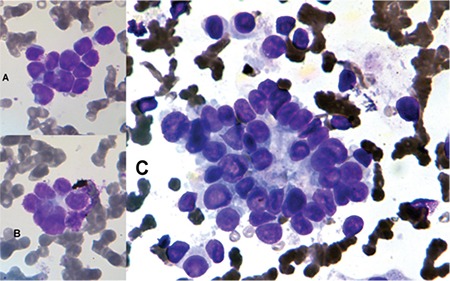
Leishman staining. A, B) Peripheral blood smear (630^x^) showing neuroblastoma tumor cell cluster with nuclear molding and rosette-like arrangement. C) Bone marrow smear (400^x^) showing Homer-Wright rosette with neuroblasts surrounding central neuropil.

A 2-year-old girl presented with fever, hepatomegaly, and progressively increasing proptosis of the right eye for 1 month. Abdominal ultrasound revealed a well-defined multi-lobulated predominantly hyperechoic mass lesion of 10.9x2.5x6.1 cm with a few foci of coarse calcification and small cystic areas arising from the right suprarenal region. The lesion was inferiorly compressing the renal parenchyma; however, the interface was well maintained. Medially it was crossing the midline and encasing the aorta and its branches. The features were of neuroblastoma. The diagnosis was confirmed by Tru-Cut biopsy from the suprarenal mass showing small round blue cells with salt and pepper chromatin. Immunohistochemistry was positive for synaptophysin. Non-contrast computerized tomography scanning of the head and orbit revealed a right retro-orbital mass with specks of calcification. The peripheral blood smears showed a few clusters and rosettes of circulating neuroblastoma cells. The bone marrow aspirate smears showed extensive infiltration by neuroblastoma cells dispersed singly, in clusters as well as in rosettes with central neuropils (Figure 1). The patient was categorized as stage IV as per the International Neuroblastoma Staging System.

Neuroblastoma is the most common extracranial solid tumor in children. The mean age of presentation is 18 months and the majority of patients (90%) are diagnosed by 5 years of age [1]. The adrenal gland is the most common primary site. Approximately 75% of children have metastases to regional lymph nodes, bone marrow, cortical bones, the orbit, the liver, and skin at the time of diagnosis. The histology shows primitive neuroblasts with variable degrees of differentiation to Schwann cells and ganglion cells. Homer-Wright rosettes, i.e. neuroblasts surrounding a tangle of neuropils, are seen. Infants may present with blue-red cutaneous masses, referred to as blueberry muffin baby. The differential diagnosis includes alveolar rhabdomyosarcoma (ARMS), Ewing’s sarcoma/primitive neuroectodermal tumor (PNET), and lymphoma. ARMS cells are more pleomorphic with abundant cytoplasm and are positive for myogenic markers (desmin, myogenin). Ewing’s sarcoma/PNET patients are usually older; cells show finely stippled chromatin- and glycogen-filled cytoplasm, with expression of CD99. Neuroblastoma may clinically mimic acute leukemia [2]. These cells can be differentiated from blasts by expression of synaptophysin or neuron-specific enolase. The blasts are positive for leukocyte common antigen (LCA/CD45). Catecholamine metabolites homovanillic acid and vanillylmandelic acid are elevated in serum and urine in approximately 95% of patients. Metastatic disease can be evaluated using iodine-123-metaiodobenzylguanidine (123-I-MIBG) scanning. N-MYC amplification is associated with poor prognosis. Morphologically identifiable circulating tumor cells (CTCs) are a rare finding [3]. Positive CTCs are associated with poor prognosis [4].
